# Physicians’ Intentions to Recommend Influenza Vaccine: A Multi-Centered Hospital-Based Study Using the Theory of Planned Behavior in Bangladesh

**DOI:** 10.3390/ijerph22010084

**Published:** 2025-01-09

**Authors:** Md Abdullah Al Jubayer Biswas, Mahbubur Rahman, Sazzad Hossain Khan, Ahamed Khairul Basher, Md Ariful Islam, Ashrak Shad Pyash, Homayra Rahman Shoshi, Md Altaf Ahmed Riaj, Md Nazrul Islam, Md Arif Rabbany, Md Azizul Haque, Shishir Ranjan Chakraborty, Syeda Rukhshana Parvin, Mahmudur Rahman, Fahmida Chowdhury, Tahmina Shirin, Md. Zakiul Hassan

**Affiliations:** 1 Programme for Emerging Infections, Infectious Disease Division, International Centre for Diarrhoeal Disease Research, Bangladesh (icddr,b), Mohakhali, Dhaka 1212, Bangladesh; sazzadhkhan1971@gmail.com (S.H.K.); khairul.basher@icddrb.org (A.K.B.); arif@icddrb.org (M.A.I.); ashrak.pyash@icddrb.org (A.S.P.); homayra.shoshi@icddrb.org (H.R.S.); ahmedriaj@icddrb.org (M.A.A.R.); fahmida_chow@icddrb.org (F.C.); zhassan@icddrb.org (M.Z.H.); 2 Collaborative Biostatistics Program, University of Saskatchewan, Saskatoon, SK S7N 5A2, Canada; 3 Institute of Epidemiology, Disease Control and Research, Mohakhali, Dhaka 1000, Bangladesh; dr_mahbub@yahoo.com (M.R.); tahmina.shirin14@gmail.com (T.S.); 4 School of Population Health, University of New South Wales, Sydney 2052, Australia; 5 Department of Neonatology, Mymensingh Medical College, Mymensingh 2200, Bangladesh; mnislamdr1@yahoo.com (M.N.I.); arifdrmmc@gmail.com (M.A.R.); 6 Department of Medicine, Rajshahi Medical College, Rajshahi 6100, Bangladesh; drazadbd@gmail.com; 7 Department of Medicine and Vice Principal, Sylhet MAG Osmani Medical College, Sylhet 3100, Bangladesh; chakrabortysrdr@gmail.com; 8 Department of Paediatrics, Khulna Medical College, Khulna 9000, Bangladesh; rumariana12@gmail.com; 9 The Eastern Mediterranean Public Health Network (EMPHNET), Bangladesh Country Office, Dhaka 1000, Bangladesh; mrahman@emphnet.net; 10 Nuffield Department of Medicine, University of Oxford, Oxford OX1 4BH, UK

**Keywords:** influenza vaccine, intention to recommend, physician attitudes and behaviors, Bangladesh, Theory of Planned Behavior (TPB)

## Abstract

Background: Influenza remains a significant public health challenge in low- and middle-income countries (LMICs) like Bangladesh, where vaccine uptake remains low despite the substantial disease burden. Physicians play a vital role in promoting vaccination, yet their intentions and influencing factors are not well understood. Methods: We conducted a cross-sectional study from June to October 2022 across four tertiary-level hospitals in Bangladesh using a questionnaire grounded in the Theory of Planned Behavior (TPB). Hierarchical logistic regression was employed to identify factors associated with vaccine recommendation intentions. Results: Among 972 physicians with an average age of 32.1 years, 40.1% intended to recommend and administer the influenza vaccine. Most (85.3%) agreed vaccination reduces risk, 65.5% desired vaccination for self-protection, 63.5% would vaccinate if available at work, and 85.3% anticipated Ministry of Health support. Male (OR = 1.9, 95% CI: 1.5–2.3) and married (OR = 1.5, 95% CI: 1.1–1.9) physicians were more likely to recommend vaccination. Each unit increase in attitude score doubled the likelihood of recommending the vaccine (OR = 2.0, 95% CI: 1.4–3.0). Conclusions: Physicians’ influenza vaccine recommendations in Bangladesh are suboptimal, influenced by gender, marital status, and attitudes. Targeted educational interventions addressing attitudinal barriers and leveraging institutional support could improve recommendation practices.

## 1. Introduction

Influenza remains a significant global health threat, contributing to substantial morbidity, mortality, and the considerable burden on healthcare systems worldwide [1,2]. Globally, annual epidemics result in an estimated 3–5 million cases of seasonal influenza-associated illness and 291,243–645,832 deaths [2,3]. Influenza also contributes significantly to hospital admissions, with an estimated 1,078,600 influenza-related acute lower respiratory infection (ALRI) hospitalizations annually [4]. The duration of influenza-related hospital stays varies by age, averaging 4.6 days for children and 5.3 days for adults, and can be up to 2.13 times longer for severe cases [5,6,7]. Such a prolonged stay contributes to the significant overall economic burden, with the cost of influenza-associated hospitalizations estimated at USD 155.1 million per season [8]. In addition to hospitalizations, influenza imposes significant costs on both outpatient and inpatient care departments of healthcare facilities [9]. For outpatient visits, including all direct and indirect expenses, the cost per episode ranges from USD 6 to USD 156 [9]. Hospitalizations add a more significant financial burden, with costs per episode ranging from USD 107 to USD 1617 [9]. Given the substantial disease and economic burdens, vaccination remains the most effective strategy to prevent influenza and its complications [3]. To mitigate the influenza-associated disease and economic burden, the World Health Organization recommends annual vaccination for high-risk groups, including children, pregnant women, the elderly, healthcare workers, and those with chronic medical conditions [10].

Despite the well-documented benefits of influenza vaccination, uptake remains low in several countries, particularly in LMICs [1]. In Bangladesh, an LMIC with over 165 million people, influenza vaccine uptake is low, leading to significant challenges in reducing the influenza disease burden [1,11,12]. As a result, influenza continues to pose a substantial burden on the population, with mortality rates of 6 per 100,000 among children under five and 41 per 100,000 among those over 60 [11]. The burden is further reflected in 4.4–6.7 hospitalizations and 100–170 outpatient visits for influenza-like illness (ILI) per 1000 people annually [11,13,14]. Approximately 25 million people visit outpatient care departments annually, costing USD 108 million, while 30,592 people are hospitalized annually with confirmed influenza, costing around USD 1.4 million [11,14]. Even among healthcare workers (HCWs), who face increased risk for both contracting and spreading the virus, vaccine uptake remains low [11,12,14]. This limited vaccination coverage heightens the vulnerability of the broader population to seasonal outbreaks and potential larger epidemics.

Healthcare providers, especially physicians, play a significant role in promoting vaccination and strongly influence their patients’ decisions to get vaccinated [15,16,17]. Physician recommendations are consistently reported as one of the potent predictors of vaccine uptake in diverse settings [16,17]. Studies support such an association, with a systematic review showing that adults who received a healthcare provider’s recommendation had 3.67 times higher odds of getting vaccinated for influenza [17]. Despite the evidence of association, many physicians do not routinely recommend influenza vaccination to their patients, even in countries with well-established vaccination programs [18,19]. In Thailand, for instance, despite a national policy to vaccinate pregnant women for influenza, only 26% of Thai physicians working in antenatal care clinics routinely recommend vaccination [18]. In contrast, in China, proactive recommendations from primary care physicians (PCPs) significantly increased influenza vaccine uptake among older adults, leading to 1100 more patients being vaccinated during the 2017–2018 flu season compared to the baseline [19]. These findings underscore the powerful impact of physician recommendations in increasing vaccine coverage.

In Bangladesh, where influenza vaccination is not part of the national immunization program, harnessing physicians’ intentions to recommend the vaccine is essential to addressing low vaccine uptake. Despite the central role of physicians in influencing vaccination behaviors, limited research exists on how Bangladeshi physicians’ attitudes and perceptions impact their intent to recommend influenza vaccines.

The Theory of Planned Behavior (TPB) provides a robust framework for investigating these intentions, having demonstrated significant predictive power in similar contexts [20,21]. For instance, a recent investigation into COVID-19 vaccine hesitancy in Bangladesh found that TPB yielded the highest predictive accuracy (adjusted R^2^ = 0.43) compared to other behavioral models [22]. Moreover, TPB has been effectively employed in other LMICs to identify critical factors influencing vaccination behaviors, including physicians’ intentions to recommend influenza vaccination [16,23,24]. Within TPB, behavioral intentions are shaped by three key dimensions: individuals’ attitudes toward the action, the influence of subjective norms, and their perceived control over the behavior [25,26]. While TPB has been widely applied to study vaccine recommendation behaviors, the majority of research has centered on high-income countries, leaving a gap in understanding how this framework applies to LMICs like Bangladesh [27,28].

Our study aimed to address this gap by applying the TPB to evaluate Bangladeshi physicians’ intentions to recommend influenza vaccines and identify the factors shaping these intentions. Understanding these determinants is crucial for developing targeted interventions to enhance influenza vaccine uptake. The findings from this study will provide insights for policymakers and public health professionals in developing effective strategies to enhance influenza vaccination coverage in Bangladesh.

## 2. Materials and Methods

### 2.1. Study Design, Setting, and Participants

We used baseline physician data from our intervention study (Trial registration: Clinicaltrials.gov NCT05521763, Version 2.0) conducted in collaboration with the Directorate General of Health Services (DGHS) of Bangladesh and received ethical approval from the Institutional Review Board of the Institute of Epidemiology, Disease Control and Research (IEDCR) [12]. The study evaluated influenza vaccination rates and explored whether increased awareness and vaccine availability could enhance uptake [12]. Conducted at four geographically distinct tertiary-level public teaching hospitals in Sylhet, Rajshahi, Khulna, and Mymensingh, Bangladesh, the baseline data were collected between June and October 2022 to minimize intervention diffusion [12]. Full details of the study design, setting, and participants have been previously published [12].

For this analysis, we focused on physicians’ baseline data, which included questions on attitudes, knowledge, and recommendation practices. These data were mapped to the Theory of Planned Behavior (TPB) framework: questions on attitudes formed the “attitude” component, perceptions of professional norms and peer influence were used for the “subjective norms” component, and knowledge of vaccine efficacy and confidence in discussing safety contributed to the “perceived control” component.

### 2.2. Sample Size and Sampling Technique

Our intervention study was a cluster randomized controlled trial, initially estimated as requiring a sample size of 435 healthcare personnel (HCPs) per arm. After adjusting for a clustering effect of 2 and allowing for a 10% non-response proportion, the adjusted sample size increased to 957 HCPs per group, resulting in a total of 3828 HCPs [12]. The full details of the sample size calculation and the sampling methodology have been provided in previous publications [12].

In our current analysis, we used a subset of 972 physicians’ data from the original dataset, without performing additional sample size recalculations or employing further sampling methods. This approach was grounded in the rigorous design of our main study, which included thorough sample size calculations and robust sampling techniques to ensure representative and reliable data. By leveraging this large baseline dataset, we investigated physicians’ attitudes, subjective norms, and perceived behavioral control concerning influenza vaccine recommendations.

### 2.3. Measures

#### 2.3.1. Study Participant Background Characteristics

Based on the literature review, we selected and included physicians’ demographic, socioeconomic, and health-related data [27,28,29,30,31,32]. Research showed that demographic factors like age, gender, marital status, type of residence, and socioeconomic indicators (e.g., income and education) might influence healthcare providers’ practices and attitudes toward recommending vaccinations [27,28,29,30,31,32]. Age in years was categorized into five groups: 18 to 25 years, 26 to 35 years, 36 to 45 years, 46 to 55 years, and above 55 years. Marital status was classified into three categories: unmarried, married, and divorced/separated/widow/widower. The type of residence was grouped into four categories: flat/apartment, house/bungalow, tin-shed roof, and others. The monthly family income in Bangladeshi Taka (BDT) was divided into two income brackets: less than 60,000 BDT and 60,000 BDT or above. Similarly, monthly family expenditure was also presented as a median and categorized into less than 50,000 BDT and 50,000 BDT or above. In terms of educational background, the highest level of education achieved was divided into post-graduation and graduation/diploma levels. Finally, participants were asked if they had any history of past illnesses or co-existing co-morbid conditions, which were classified as either yes or no.

#### 2.3.2. Attitude Toward the Behavior

**Figure 1:**
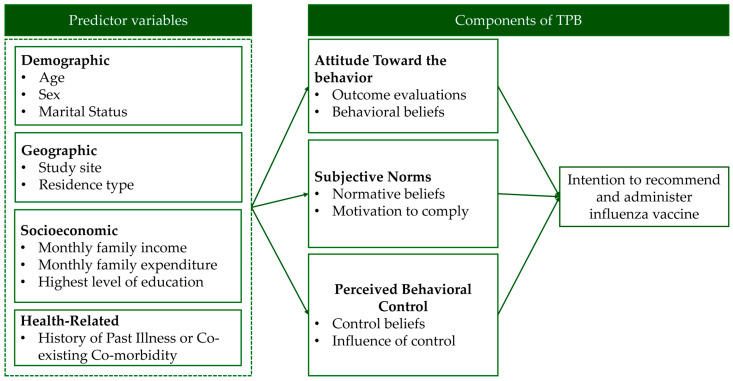
TPB framework to investigate physicians’ intention to recommend and administer influenza vaccine.

**Figure 2:**
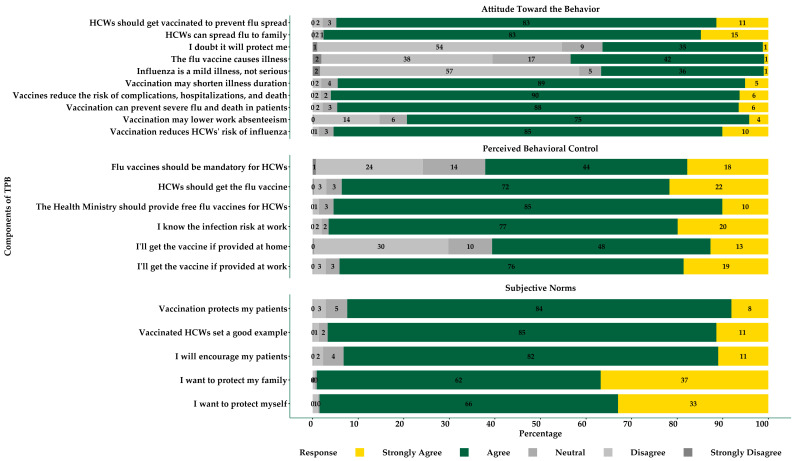
Physicians’ responses across TPB-component-related questions for influenza vaccination attitudes, subjective norms, and perceived behavioral control.

Attitude was evaluated using ten questions, with responses measured on a 5-point Likert scale spanning options from “strongly disagree” to “strongly agree”. Ten questions were categorized into outcome assessment and behavioral beliefs, which were components in understanding physicians’ attitudes toward influenza vaccine recommendation. Under the outcome evaluation domain, questions included “Vaccination reduces HCWs’ risk of influenza”, “Vaccination may lower work absenteeism”, “Vaccination can prevent severe flu and death in patients”, and “Vaccines reduce the risk of complications, hospitalizations, and death”. Other questions addressed the perception that vaccination could shorten the duration of illness and that HCWs should get vaccinated to prevent the spread of influenza to others, including family members. The behavioral belief domain captured personal beliefs and misconceptions about influenza and the vaccine itself. Questions in this domain included “Influenza is a mild illness, not serious” and “The flu vaccine causes illness”, both of which reflect common misconceptions. Additionally, beliefs such as “I doubt it will protect me” and concerns about spreading the flu to family members were included to assess how these factors influence vaccination behavior. Outcome evaluations and behavioral beliefs constructed components of physicians’ attitudes toward influenza vaccination (Figure 1 and Figure 2).

#### 2.3.3. Subjective Norms

Subjective norms were evaluated through five questions to assess the perceived social pressure to vaccinate, with responses captured on a 5-point Likert scale. These questions were categorized into two key domains: normative beliefs and motivation to comply. The normative beliefs domain focused on the influence of social expectations regarding vaccination. Questions included “I want to protect myself”, “I want to protect my family”, and “I will encourage my patients”. In contrast, the motivation to comply domain focused on how compelled physicians were to meet these social and professional expectations. Questions such as “Vaccinated HCWs set a good example” and “Vaccination protects my patients” assessed the degree to which physicians were motivated to align their behavior with perceived social norms, particularly regarding the example they set for others and their role in patient safety (Figure 1 and Figure 2).

#### 2.3.4. Perceived Behavioral Control

Perceived behavioral control was assessed using six questions that evaluated physicians’ perceptions of the difficulty of vaccinating, with responses recorded on a 5-point Likert scale. These questions were divided into two key domains: control beliefs and influence of control. The control beliefs domain included questions like “I’ll get the vaccine if provided at work” and “I’ll get the vaccine if provided at home”, reflecting the ease of accessing the vaccine. Another question, “I know the infection risk at work”, reflected physicians’ awareness of workplace risks that might influence their decision to vaccinate. In the influence of control domain, the focus was on the external factors and policies that could impact physicians’ behavior. Questions such as “The Health Ministry should provide free flu vaccines for HCWs”, “HCWs should get the flu vaccine”, and “Flu vaccines should be mandatory for HCWs” explored the perceived influence of institutional policies and expectations on vaccination behavior (Figure 1 and Figure 2).

#### 2.3.5. Intention to Perform Behavior

Intention to recommend and administer the influenza vaccine was recorded using a binary variable with a “yes” or “no” response. The question asked physicians, “If the influenza vaccine was available for your patients, would you vaccinate them or recommend receiving the vaccine?” This variable captured the physicians’ direct intention to either administer or recommend the influenza vaccine to their patients, also indicating their willingness to promote vaccination in clinical practice (Figure 1 and Figure 2).

### 2.4. Statistical Analysis

We used descriptive statistical tools to present the physicians’ background characteristics and responses within the domains of the TPB. For categorical variables, we used cross-tabulations. Continuous variables were presented using means, standard deviations (SD), medians, and percentiles. Before the multivariable analysis, we assessed the internal consistency of each TPB domain using Cronbach’s alpha. The reliability coefficients for attitudes (α = 0.6257), subjective norms (α = 0.6738), and perceived behavioral control (α = 0.6618) were found to be acceptable, even if slightly below the conventional threshold of 0.70 (Tables S1 and S2). While 0.70 is often cited as a minimally acceptable value, some evidence found that alpha values between 0.60 and 0.70 could be deemed acceptable, particularly in exploratory research or when dealing with psychological constructs [28,33,34,35]. We also assessed the construct validity of the TPB utilizing Principal Component Analysis (PCA) with varimax rotation, we used the Kaiser–Meyer–Olkin (KMO) measure of sampling adequacy, scree plot analysis, and factor loadings greater than 0.40 to identify how many factors should be retained [34,35,36,37]. We found a high value of 0.851 in the KMO test, indicating that the data were appropriate for factor analysis [28,33,34,35,36,37]. We carried out a hierarchical logistic regression analysis to explore the TPB components’ association with physicians’ vaccine recommendation intention, accounting for their background characteristics. Our analysis had a two-step hierarchical structure. The first model incorporated background characteristic variables as control factors. Background characteristic variables (age, sex, marital status, and study site) that were significant at the 10% level were included in the first multivariable model. The second model incorporated the TPB constructs, including attitudes, subjective norms, and perceived behavioral control. The results of both models were expressed as an adjusted odds ratio (OR) with a 95% confidence interval. Statistical analyses were conducted using two-sided hypothesis tests, with significance determined at *p*-values below 0.05. To determine the incremental predictive value of the TPB variables beyond demographic factors, model comparisons were made using Akaike’s information criterion (AIC), the Bayesian information criterion (BIC), and the area under the curve (AUC) from receiver operating characteristic (ROC) analysis. The second model showed an improved fit with a lower AIC value and a higher AUC, although its BIC value was slightly higher than that of the first model. Model fit was further evaluated using the Hosmer–Lemeshow test. All analyses were carried out using SAS version 9.4 (SAS Institute Inc., Cary, NC, USA).

## 3. Results

### 3.1. Study Participant Background Characteristics

We analyzed data from 972 physicians. The average age was 32 years (SD = 7.6), with a median age of 30 (IQR: 36–26). Most physicians (58%) were between 26 and 35 years old, while 1.3% were in the over-55 age group. Concerning sex, 55% of the sample was male. The median monthly family income was 60,000 BDT (75th–25th percentile: 100,000–40,000 BDT), with 54% earning ≥ 60,000 BDT and 46% earning less. Monthly family expenditure had a median of 50,000 BDT (75th–25th percentile: 70,000–30,000 BDT), with 55% spending ≥50,000 BDT and 45% spending less. For education, 69% of respondents had completed graduation (including a diploma), while 31% had pursued post-graduate studies (Table 1).

### 3.2. Physician Attitudes, Social Norms, and Behavioral Control over the Intention to Recommend the Influenza Vaccine 

We found that most physicians strongly agreed or agreed with positive beliefs about vaccination. Specifically, 85% agreed that vaccination reduces the risk of influenza for HCWs, while 90% believed it reduces the risk of complications, hospitalizations, and death. Likewise, 88% agreed that vaccination can prevent severe flu and death in patients. Misconceptions were less common, with 57% disagreeing that influenza is a mild illness and 54% disagreeing that the flu vaccine is ineffective for personal protection. However, 42% still believed the misconception that the flu vaccine causes illness. Regarding subjective norms, 65% of respondents agreed that they wanted to protect themselves, and 62% wanted to protect their families. Furthermore, 82% reported encouraging their patients to get vaccinated, and 85% felt vaccinated HCWs set a good example for others. A high proportion (84%) agreed that vaccination helps protect their patients from flu. Regarding perceived behavioral control, 75% of respondents reported they would get vaccinated if it was provided at work. Additionally, 85% of respondents agreed that the Ministry of Health should provide free flu vaccines for HCWs, and 72% believed HCWs should get vaccinated. However, there were mixed feelings about mandatory vaccination policies, with 44% agreeing that flu vaccines should be mandatory for HCWs, while 23% disagreed (Figure 2).

### 3.3. Intention to Recommend and Administer the Influenza Vaccine

In our analysis, among 972 physicians, 40% of physicians reported an intention to recommend and administer the influenza vaccine. The highest proportion of physicians intending to recommend the influenza vaccine were aged 26–35 (58%), while the lowest were those over 55 (0.5%), with a significant association between age and intention (*p* = 0.035). Additionally, sex differences were significant, with a higher proportion of males intending to recommend the vaccine (62% vs. 50%, *p* = 0.001). Marital status was also found to have a significant association, as a higher proportion of married physicians reported recommending the vaccine than unmarried physicians (72% vs. 28%, *p* = 0.001). The healthcare facility where the physicians worked was also a significantly associated factor, with the highest proportion of vaccine recommendations occurring at Healthcare Facility 4 (36%) and the lowest at Healthcare Facility 3 (36% vs. 11.9%, *p* < 0.0001). On the contrary, several factors were not significantly associated with the intention to recommend and administer the influenza vaccine. Non-significant factors included residence type (*p* = 0.118), monthly family income (*p* = 0.552), monthly family expenditure (*p* = 0.295), and education level (*p* = 0.437) (Table 1).

### 3.4. Factors Associated with Physicians’ Attitudes, Social Norms, and Behavioral Control over the Intention to Recommend Influenza Vaccination

Table 2 presents the hierarchical logistic regression analysis assessing sociodemographic factors and TPB components’ association with physicians’ intentions to recommend and administer the influenza vaccine. In Model 1, we found that male physicians had a higher likelihood (OR = 1.9, 95% CI: 1.5–2.3) of recommending the vaccine than female physicians. Married physicians were 1.5 times (OR = 1.5, 95% CI: 1.1–1.9) more likely to recommend the vaccine in comparison with those who were unmarried. Regarding the study site, physicians from Healthcare Facility 1 were 1.7 times (OR = 1.7, 95% CI: 1.3–2.2) more prone to recommend the vaccine than those from Healthcare Facility 4. In Model 2, after adding TPB components, the odds ratios for sex, marital status, and study site remained almost unchanged, indicating that these sociodemographic factors played a significant role even after adjusting for TPB components. We found that physicians with more positive attitudes toward vaccination had increased odds of recommending the vaccine, with each unit increase in attitude score associated with a two-fold rise in the probability of recommending the influenza vaccine (OR = 2.0, 95% CI: 1.4–3.0). However, we found no significant association between subjective norms and perceived behavioral control.

## 4. Discussion

Our study reveals a concerningly low proportion of physicians in Bangladesh—only 40.1%—who intend to recommend and administer the influenza vaccine. This figure is notably lower than those reported in some low- and middle-income countries (LMICs) and significantly below rates observed in high-income nations. For instance, studies have shown that in Pakistan, 26.0% of healthcare professionals frequently recommend influenza vaccines to patients, while in India and Lebanon, the proportions are 82.8% and 76.8%, respectively [38,39,40]. In contrast, high-income countries like the USA and Saudi Arabia reported 82% and 63.7% recommendation rates, respectively [41,42]. These disparities underscore a critical gap in vaccine advocacy among Bangladeshi physicians, highlighting the urgent need for targeted interventions to enhance vaccine recommendation practices within this demographic.

A critical finding of our study is the pivotal role of attitudes in shaping physicians’ intention to recommend the vaccine. Physicians with more positive attitudes were twice as likely to recommend the influenza vaccine, a result consistent with TPB-based studies globally. For example, research from South Korea and Hong Kong similarly found attitudes to be the strongest predictors of vaccination intent [24,43]. This reinforces the need for targeted educational campaigns to enhance physicians’ understanding of the benefits of influenza vaccination, addressing both clinical efficacy and the broader public health impact. By fostering positive attitudes, healthcare institutions can bridge the gap between physicians’ knowledge and their actual recommendation practices [44].

Our analysis also revealed a significant gender disparity, with male physicians being twice as likely as their female counterparts to recommend the vaccine. This contrasts with some studies where female physicians were more proactive in promoting vaccinations [45,46]. Cultural and systemic factors in Bangladesh, such as differing professional responsibilities or exposure to vaccination advocacy programs, may account for this discrepancy. Tailored strategies addressing gender-specific barriers could help equalize recommendation practices and enhance overall vaccination rates.

Marital status emerged as another influential factor, with married physicians showing a significantly higher likelihood of recommending the vaccine than unmarried peers. This could reflect a sense of heightened responsibility toward family and community health among married individuals [30,47]. Additionally, the notable differences in vaccine recommendation intentions across healthcare facilities highlight the role of institutional policies and environments [48]. Standardizing vaccination promotion strategies and ensuring uniform availability of vaccines across facilities may reduce these disparities and create a more cohesive public health approach.

Despite strong positive beliefs about vaccination benefits, our findings revealed persistent misconceptions, with 42.5% of physicians believing that the flu vaccine causes illness. This indicates a critical barrier to vaccine promotion that needs to be addressed through myth-busting campaigns and evidence-based training sessions. Given the high proportion of physicians (85.3%) supporting free vaccines provided by the Ministry of Health, structural interventions, such as workplace vaccination programs, could further strengthen physicians’ intent to recommend and administer the vaccine.

Our study leverages the TPB framework to provide insights into vaccine recommendation practices’ psychological and social drivers. The geographically diverse sample enhances the generalizability of our findings within Bangladesh. However, the study’s cross-sectional design makes it arduous to determine causal relationships, while the use of self-reported data raises the possibility of response bias [49]. Future research should incorporate longitudinal approaches and observational data to validate these findings and explore changes in physicians’ attitudes and behaviors over time.

## 5. Conclusions

Our study underscores the need for targeted interventions to address the low influenza vaccine recommendation rates among Bangladeshi physicians. Enhancing physicians’ attitudes through education, dispelling misconceptions, and implementing structural supports like free workplace vaccination programs could significantly improve vaccine uptake. These findings provide a roadmap for public health strategies aimed at empowering physicians to become more active advocates for influenza prevention in Bangladesh.

## Figures and Tables

**Table 1:** Sociodemographic characteristics and intention to recommend and administer influenza vaccine among physicians, Bangladesh, June and October 2022.

Intention to Recommend and Administer Influenza Vaccine				
	**Total**	**No**	**Yes**	
**Variables**	**n (col%)**	**n (col%)**	**n (col%)**	***p*-Value**
**No. of respondents**	**972 (100)**	**584 (100)**	**388 (100)**	
**Age in years**				
Mean ± SD	32.1 ± 7.6	31.7 ± 7.9	32.7 ± 7.2	
Median (75th–25th percentile)	30 (36–26)	28 (36–26)	32 (36–26)	
18–25	96 (9.9)	68 (11.7)	28 (7.2)	0.035
26–35	563 (57.9)	337 (57.7)	226 (58.2)	
36–45	249 (25.6)	141 (24.1)	108 (27.8)	
46–55	51 (5.2)	27 (4.6)	24 (6.2)	
>55	13 (1.3)	11 (1.9)	2 (0.5)	
**Sex**				
Male	533 (54.8)	294 (50.3)	239 (61.6)	0.001
Female	439 (45.2)	290 (49.7)	149 (38.4)	
**Marital status**				
Unmarried	335 (34.5)	227 (38.9)	108 (27.8)	0.001
Married	634 (65.2)	355 (60.8)	279 (71.9)	
Divorced/Separated/Widow/Widower	3 (0.3)	2 (0.3)	1 (0.3)	
**Study Site**				
Healthcare facility 1 (Khulna)	224 (23)	120 (20.6)	104 (26.8)	<0.0001
Healthcare facility 2 (Mymensingh)	239 (24.6)	140 (24.0)	99 (25.5)	
Healthcare facility 3 (Sylhet)	210 (21.6)	164 (28.0)	46 (11.9)	
Healthcare facility 4 (Rajshahi)	299 (30.8)	160 (27.4)	139 (35.8)	
**Residence type**				
Flat/apartment	639 (65.7)	371 (63.5)	268 (69.1)	0.118
House/bungalow	302 (31.1)	189 (32.4)	113 (29.1)	
Tin shed roof	7 (0.7)	6 (1.0)	1 (0.3)	
Others	24 (2.5)	18 (3.1)	6 (1.5)	
**Monthly family income**				
Median (75th–25th percentile)	60,000 (100,000–40,000)	60,000 (100,000–40,000)	60,000 (100,000–45,000)	
<60,000	443 (45.6)	271 (46.4)	172 (44.3)	0.552
≥60,000	529 (54.4)	313 (53.6)	216 (55.7)	
**Monthly family expenditure**				
Median (75th–25th percentile)	50,000 (70,000–30,000)	50,000 (70,000–30,000)	50,000 (70,000–40,000)	
<50,000	436 (44.9)	274 (46.9)	162 (41.8)	0.295
≥50,000	536 (55.1)	310 (53.1)	226 (58.2)	
**Highest level of education**				
Post-graduation	303 (31.2)	177 (30.3)	126 (32.5)	0.437
Graduation including diploma	669 (68.8)	407 (69.7)	262 (67.5)	
**Having any history of past illness or co-existing co-morbid conditions**				
Yes	205 (21.1)	465 (79.6)	302 (77.8)	0.522
No	767 (78.9)	119 (20.4)	86 (22.2)	

**Table 2:** Hierarchical logistic regression analysis to investigate the association between physicians’ intention to recommend the influenza vaccine and components of the TPB.

Variables	Model 1	Model 2
	OR (95% CI)	OR (95% CI)
**Age in years**		
18–25 ***	2.0 (0.7–6.4)	2.0 (0.6–6.5)
26–35	2.9 (1–8.7)	3.0 (1.0–9.0)
36–45	2.3 (0.8–6.9)	2.4 (0.8–7.3)
46–55	2.2 (0.7–6.9)	2.2 (0.7–6.9)
>55	1	1
**Sex**		
Male	1.9 (1.5–2.3) ***	1.9 (1.6–2.3) ***
Female	1	1
**Marital status**		
Married	1.5 (1.1–1.9) **	1.5 (1.2–1.9) **
Divorced/Separated/Widow/Widower	2.5 (0.4–16.4)	2.7 (0.4–17.4)
Unmarried	1	1
**Study Site**		
Healthcare facility 1	1.7 (1.3–2.2) ***	1.5 (1.1–1.9) ***
Healthcare facility 2	0.2 (0.2–0.3) ***	0.2 (0.1–0.3) **
Healthcare facility 3	1.1 (0.9–1.4)	1.0 (0.7–1.3)
Healthcare facility 4	1	1
**Components of the TPB**		
Attitude toward the behavior		2.0 (1.4–3.0) ***
Subjective norms		1.2 (0.8–1.7)
Perceived behavioral control		0.9 (0.6–1.2)
**Model performance indicators**		
Akaike’s information criterion	2379	2366.4
Bayesian information criterion	2440.2	2444.3
Hosmer–Lemeshow goodness-of-fit test	𝒳^2^ = 10.81, *p*-value = 0.2127	𝒳^2^ = 14.86, *p*-value = 0.0.0619
Area under ROC	0.6919	0.7052

** *p*-value < 0.01; *** *p*-value < 0.001.

## Data Availability

The datasets presented in this article are not readily available because of technical limitations. Requests to access the datasets should be directed to M.Z.H.

## Supplementary Materials

The following supporting information can be downloaded at https://www.mdpi.com/article/10.3390/ijerph22010084/s1, Table S1: Internal consistency of components of the Theory of Planned Behavior (TPB) among physicians in Bangladesh, June–October 2022; Table S2: Score of attitude toward behavior, subjective norms, and perceived behavioral control among physicians in Bangladesh, June–October 2022.
