# Supplementary material for: Physicians’ Intentions to Recommend Influenza Vaccine: A Multi-Centered Hospital-Based Study Using the Theory of Planned Behavior in Bangladesh
Source: Int J Environ Res Public Health. 2025 Jan 9;22(1):84. doi: 10.3390/ijerph22010084 (PMC11765029; doi:10.3390/ijerph22010084)
Supplement: Supplementary file 1 [file ijerph-22-00084-s001.zip › Table S1.pdf]

Supplementary Table S1 : Internal consistency of components of the Theory of Planned Behavior (TPB) among Physicians in Bangladesh, June-October 2022

Components of TPB	Range (Min, Max)	Mean ± SD	Internal consistency (Cronbach's alpha)
**Attitude Toward the Behavior (Personal beliefs about vaccination and its consequences)**			
Vaccination reduces HCWs' risk of influenza	(1,5)	4.0 ± 0.4	0.6257
Vaccination may lower work absenteeism	(1,5)	3.7 ± 0.8	
Vaccination can prevent severe flu and death in patients	(1,5)	4.0 ± 0.4	
Vaccines reduce the risk of complications, hospitalizations, and death	(1,5)	4.0 ± 0.4	
Vaccination may shorten illness duration	(1,5)	4.0 ± 0.4	
Influenza is a mild illness, not serious	(1,5)	3.2 ± 1.0	
The flu vaccine causes illness	(1,5)	3.0 ± 1.0	
I doubt it will protect me	(1,5)	3.2 ± 1.0	
HCWs can spread flu to family	(1,5)	4.1 ± 0.5	
HCWs should get vaccinated to prevent flu spread	(1,5)	4.0 ± 0.5	
**Subjective Norms (Perceived social pressure to vaccinate)**			
I want to protect myself	(1,5)	4.3 ± 0.5	0.6738
I want to protect my family	(1,5)	4.4 ± 0.5	
I will encourage my patients	(1,5)	4.0 ± 0.5	
Vaccinated HCWs set a good example	(1,5)	4.1 ± 0.4	
Vaccination protects my patients	(1,5)	4.0 ± 0.5	
**Perceived Behavioral Control (Perception of ease or difficulty in getting vaccinated)**			
I'll get the vaccine if provided at work	(1,5)	4.1 ± 0.6	0.6618
I'll get the vaccine if provided at home	(1,5)	3.4 ± 1.1	
I know the infection risk at work	(1,5)	4.1 ± 0.5	
The Health Ministry should provide free flu vaccines for HCWs.	(1,5)	1.5 ± 0.6	
HCWs should get the flu vaccine	(1,5)	1.9 ± 0.6	
Flu vaccines should be mandatory for HCWs	(1,5)	2.4 ± 1.5	
