# Supplementary material for: Physicians’ Intentions to Recommend Influenza Vaccine: A Multi-Centered Hospital-Based Study Using the Theory of Planned Behavior in Bangladesh
Source: Int J Environ Res Public Health. 2025 Jan 9;22(1):84. doi: 10.3390/ijerph22010084 (PMC11765029; doi:10.3390/ijerph22010084)
Supplement: Supplementary file 1 [file ijerph-22-00084-s001.zip › Table S2.pdf]

Supplementary Table S2 : Score of attitude toward behavior, subjective norms, and perceived behavioral control among physicians in Bangladesh, June-October 2022

Variables	Attitude Toward the behavior	Subjective Norms	Perceived Behavioral Control
	**Mean ± SD**	**Mean ± SD**	**Mean ± SD**
Overall	3.7 ± 0.3	3.9 ± 0.3	2.9 ± 0.3
**Age in year**			
18-25***	3.7 ± 0.3	4.0 ± 0.3	2.9 ± 0.3
26-35	3.7 ± 0.3	3.9 ± 0.3	2.9 ± 0.3
36-45	3.7 ± 0.3	3.8 ± 0.4	2.9 ± 0.3
46-55	3.7 ± 0.3	3.9 ± 0.4	3.0 ± 0.3
>55	3.6 ± 0.3	3.8 ± 0.4	2.9 ± 0.4
**Sex**			
Male	3.7 ± 0.3	3.9 ± 0.4	2.9 ± 0.3
Female	3.7 ± 0.3	3.9 ± 0.3	2.9 ± 0.3
**Marital status**			
Unmarried	3.7 ± 0.3	4.0 ± 0.4	2.9 ± 0.3
Married	3.7 ± 0.3	3.8 ± 0.3	2.9 ± 0.3
Divorced/Separated/Widow/Widower	3.6 ± 0.2	3.9 ± 0.3	2.9 ± 0.3
**Study Site**			
Healthcare facility 1	3.6 ± 0.2	3.9 ± 0.3	3.1 ± 0.3
Healthcare facility 2	3.7 ± 0.3	4.0 ± 0.3	2.8 ± 0.3
Healthcare facility 3	3.7 ± 0.3	3.9 ± 0.3	2.9 ± 0.3
Healthcare facility 4	3.8 ± 0.2	3.8 ± 0.4	2.9 ± 0.3
**Residence type**			
Flat/apartment	3.7 ± 0.3	3.9 ± 0.3	2.9 ± 0.3
House/Bunglow	3.8 ± 0.3	3.9 ± 0.4	2.9 ± 0.3
Tin shed roof	3.6 ± 0.4	4.0 ± 0.5	2.9 ± 0.2
Others	3.8 ± 0.2	4.1 ± 0.2	2.7 ± 0.2
**Height level of education**			
Post-graduation	3.7 ± 0.3	3.8 ± 0.4	2.9 ± 0.3
Graduation including diploma	3.8 ± 0.3	3.9 ± 0.3	2.9 ± 0.3
**Having any history of past illness or co-existing co-morbid conditions**			
Yes	3.7 ± 0.3	3.9 ± 0.3	2.9 ± 0.3
No	3.7 ± 0.3	3.9 ± 0.4	2.9 ± 0.4
